# Evaluation of Four Different Adhesive Systems’ Bonding Strength Between Superficial and Deep Dentin

**DOI:** 10.3390/ma18133107

**Published:** 2025-07-01

**Authors:** Dersim Gökce, Aslihan Usumez, Zelal Seyfioglu Polat, Emrah Ayna

**Affiliations:** 1 Department of Prosthodontics, Faculty of Dentistry, İstanbul Atlas University, İstanbul 34403, Turkey; 2 İstanbul Dental Academy, İstanbul 34015, Turkey; 3 Department of Prosthodontics, Faculty of Dentistry, Dicle University, Diyarbakır 21280, Turkey

**Keywords:** adhesive, superficial dentin, deep dentin, bonding, dental materials

## Abstract

The success of adhesive restorations largely depends on the optimal bond strength between the tooth structure and the restorative material. The aim of this study was to evaluate the shear bond strength (SBS) of four different adhesives applied to mandibular molars on deep and superficial dentin. The total of 56 teeth used in the study were randomly divided into 2 subgroups of superficial dentin and deep dentin participants (n = 28). Superficial and deep dentin groups were randomly divided into 4 subgroups (n = 7) for application with different adhesive agents. We formed the following groups: Group 1 (G1)—deep dentin and GC bonding agent (G-Premio BOND); Group 2 (G2)—superficial dentin and GC bonding agent; Group 3 (G3)—deep dentin and Clearfil S3 bond bonding agent (Clearfil TM S3 BOND); Group 4 (G4)—superficial dentin and Clearfil S3 bond bonding agent; Group 5 (G5)—deep dentin and KerrOptibond bonding agent (KerrOptibond^TM^ Universal); Group 6 (G6)—superficial dentin and Kerr Optibond bonding agent; Group 7 (G7)—deep dentin and 3M-ESPE universal bonding agent (3M ESPE); Group 8 (G8)—superficial dentin and 3M-ESPE universal bonding agent. The silicone block with a diameter of 3 mm and a thickness of 1 mm was placed in the middle of the occlusal surface and the test composite was loaded. All prepared specimens were aged in thermal cycles at 5–55 °C for 5000 cycles. The teeth were subjected to SBS (shear bond strength) tests at a crosshead speed of 1 mm/min in a universal testing machine. In all adhesive systems, deep dentin showed a higher bond strength than superficial dentin and the bond strength value was statistically significant (*p* = 0.05). The bond strength in all tested adhesive systems was observed to be significantly higher in deep dentin than in superficial dentin.

## 1. Introduction

In recent years, the increasing patient demand for cosmetic, conservative restorations has led to a significant surge in the use of direct composite restorations. Adhesive restoration, directly linked to dental components in both anterior and posterior teeth, is frequently utilized [1,2]. The effectiveness of adhesive restorations fundamentally depends on the optimal bonding strength between the tooth substrate and the restorative material. Exemplary restoration must guarantee optimal marginal adaptation, enhance tooth structure, and exhibit long-term durability [2].

Dentin tissue is a continual challenge for adhesion using bonding techniques due to its complexity and dynamic characteristics [3,4]. Dentin tissue is characterized as a biological composite consisting of a collagen matrix permeated by parallel micrometer-sized hypermineralized apatite crystallites. Dentinal tubules lack collagen and are composed of peritubular dentin [4,5]. The chemical composition of dentin tissue generally consists of 50% minerals, 20% water, and 30% organic matrix; however, its composition may change under different conditions. This is because superficial dentin contains a restricted quantity of tubules and is predominantly made up of intertubular dentin. Deep dentin, situated adjacent to the pulp, primarily exhibits bigger funnel-shaped dentinal tubules and significantly diminished intertubular dentin [4,6].

Both the structure of dentin and the variation in water content relative to dentin depth must be considered. The substantial water content of dentin is confined to dentinal tubules, with the density of these tubules fluctuating with dentin depth; hence, water content is anticipated to be greater in deep dentin and lesser in superficial dentin [4,7]. This condition is crucial for assessing modern adhesive systems classified as “E and R (total etch)” and “self-etch” according to their adhesion techniques. Nevertheless, it is an essential consideration in the utilization of “strong” (pH < 1), moderately strong (pH~1.5), or “mild” self-etch adhesives (pH~2) in relation to their acidity [4,8].

In total etch adhesive systems, the initial step involves applying acid to enamel and dentin to completely remove the smear layer, therefore exposing the collagen fibrils in the dentin and increasing surface energy [9]. The second stage involves the application of primers that act as adhesion promoters, consisting of monomers with hydrophobic properties for copolymerization with the adhesive resin and displaying hydrophilic attributes that attract exposed collagen fibrils [10]. The principal function of the primer is to transform the hydrophilic dentin surface into a hydrophobic one, hence enhancing the adhesive’s penetration into the collagen fiber matrix [11]. The total etch technique presents a risk of collagen fiber degradation during drying, blocking interfibrillar spaces and impeding adhesive penetration [12]. Insufficient adhesive penetration in demineralized dentin may result in exposed collagen at the dentin–adhesive interface [13], potentially allowing bacterial infiltration and undermining the bond’s stability [14].

This in vitro study looked to assess the shear bond strengths of four different adhesives applied to mandibular molars, employing a total etch adhesive system on both deep and superficial dentin, while acknowledging the significance of bonding strategies, composition, acidity, and solvents in adhesive systems and their impact on bond strength across various tooth regions.

Universal or multimodal adhesives constitute a new class of single-step adhesives, distinguished by two main attributes: first, their suitability for composite restorations requiring adhesion to various substrates, especially heterogeneous ones; second, their adaptability for use in self-etch (SE), selective etch, or etch and rinse (E and R) techniques, depending on the clinician’s choice [1,15].

Universal adhesives may contain 10 methacryloyloxydecyl dihydrogen phosphate (10 MDP), glycero-phosphate dimethacrylate (GPDM), and amide monomer, enhancing their durability and facilitating the formation of a hydrolysis-resistant interface (hybrid layer) over time [16,17,18]. However, due to their recent introduction to the market, there is no consensus about the advantages of one particular etching mode (SE or E and R) over the other in the context of universal adhesives [1]. Evidence suggests that using SE universal adhesives in E and R mode does not adversely affect primary bond strength or fatigue resistance [1]. In contrast, Clearfil SE Bond, a self-etching adhesive, exhibits significantly decreased shear bond strength and fatigue resistance when employed in E and R mode [1]. The SBS test is widely utilized to evaluate the bond strength of different dentin bonding agents [19]. 

The hypotheses investigated were as follows: (1) dentin adhesives demonstrate comparable bonding efficacy to superficial and deep dentin; (2) there is no difference in shear bond strength among several universal adhesives applied via the etch and rinse (E and R) method to superficial and deep dentin.

## 2. Materials and Methods

The study utilized 56 caries-free, unrestored, and uncracked mandibular molars. Following the collection of the teeth, they were immersed in a 0.1% thymol solution at 4 °C for disinfection. The teeth were preserved in a 0.9% saline solution until the initiation of the examination. Soft tissue remnants and dental calculus were removed utilizing a scaler and ultrasonic device (Ca-vitron). The teeth were randomly assigned to two subgroups: superficial dentin and deep dentin (n = 28). The occlusal surfaces of the teeth were abraded. The superficial dentin group was established following the thorough removal of the occlusal enamel from the teeth with a fissure bur (GZ, GZ Instrumente Gmbh, Lustensu, Austria) under water cooling, thereby revealing the dentin tissue (4–5 mm coronal to the cemento-enamel junction). In the deep dentin group, the dentin tissue was configured with a fissure bur under water cooling, preserving 0.5 mm of dentin above the pulp chamber. The procedure was halted upon the observation of the pulp chamber’s shadow. The enamel on the occlusal surface of the teeth was entirely abraded using 180-grit sandpaper and water chilling. The superficial and deep dentin groups were randomly divided into four subgroups (n = 7) for treatment with different adhesive agents. Orthophosphoric acid (K-etchantsyringe, Clearfil Majesty, Kuraray, Okayama, Japan) was employed in all groups, utilizing a total etch technique on the dentin for 15 s. The engraved teeth were subjected to an air–water spray for 10 s and then dried with an air spray for 5 s.

**Group 1 (G1):** A GC bonding agent (G-Premio BOND, GC Corporation, Tokyo, Japan) was applied to deep dentin.

**Group 2(G2):** A GC bonding agent (G-Premio BOND, GC Corporation, Tokyo, Japan) was applied to superficial dentin.

**Group 3 (G3):** A Clearfil S3 bond bonding agent (Clearfil TM S3 BOND Universal Kit, Kuraray, Okayama, Japan) was applied to deep dentin.

**Group 4 (G4):** A Clearfil S3 bond bonding agent (Clearfil TM S3 BOND Universal Kit, Kuraray, Okayama, Japan) was applied to superficial dentin.

**Group 5 (G5):** A Kerr Optibond bonding agent (KerrOptibond^TM^ Universal, Kerr Corporation, Orange, CA, USA) was applied to deep dentin.

**Group 6 (G6):** A Kerr Optibond bonding agent (KerrOptibond^TM^ Universal, Kerr Corporation, Orange, CA, USA) was applied to superficial dentin.

**Group 7 (G7):** A 3M-ESPE (Single Bond Universal) universal bonding agent (3M ESPE, St.Paul, MN, USA) was applied to deep dentin.

**Group 8 (G8):** A 3M-ESPE (Single Bond Universal) universal bonding agent (3M ESPE, St.Paul, MN, USA) was applied to superficial dentin (Table 1).

The adhesive agent was diluted via air spray application and subsequently reapplied to the designated dentin surface with a brush and further diluted. The polymerization was conducted using an LED light apparatus (Woodpecker Medical Instrument Co., Ltd., Guilin, China) for a duration of 20 s at an intensity of 2300 Mw/cm^2^.

A silicone block (Elite HD+, Zhermack, Italy) with a diameter of 3 mm and a thickness of 1 mm was positioned on the central occlusal surface of the tooth, and the experimental composite (BioInfinity Sirius Universal Dental Composite, Avrupa İmplant (Umg Uysal), Istanbul, Turkey) was applied to the dentin surface utilizing the silicone block.

**Figure 1:**
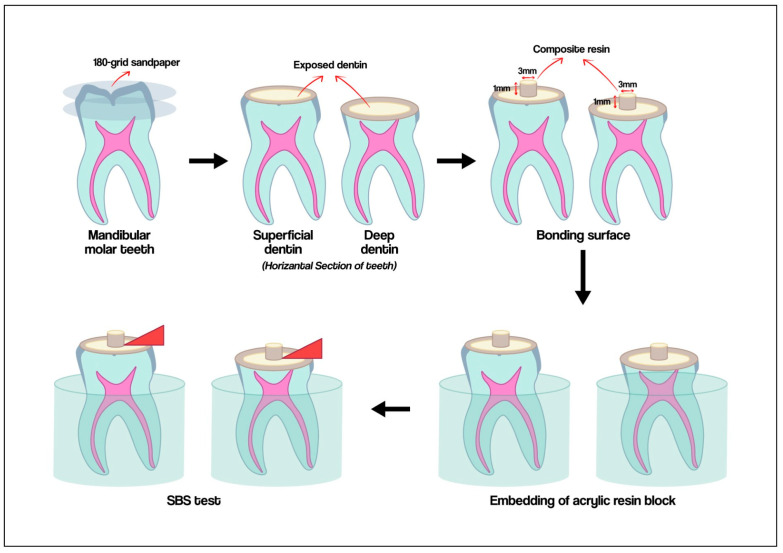
The schematic picture of specimen preparation.

The experimental composite was polymerized using an LED light device (Woodpecker Medical Instrument Co., Ltd., Guilin, China) for a duration of 20 s. All prepared specimens underwent thermal cycling at 5–55 °C (MOD Dental, Esetron Smart Robotechnologies, Ankara, Turkey) for 5000 cycles. Molars were then embedded in molds with a diameter of 2.5 cm and a length of 3 cm, which were subsequently filled with self-curing acrylic resin. The specimens underwent a shear bond strength (SBS) test at a crosshead speed of 1 mm/min utilizing universal testing equipment (Tensile/Shear, MOD Dental, Esetron Smart Robotechnologies, Ankara, Turkey). A load was applied to the composite–tooth interface at a crosshead speed of 1 mm/min until failure ensued. The peak load displayed on the monitor at fracture (in Newtons) was documented and divided by the cross-sectional area of the composite cylinder (in square millimeters) to determine the shear bond strength (SBS) value in megapascals (MPa). The failure mode was discovered and categorized as adhesive (debonding at the interface), cohesive (debonding within the tooth structure or composite mass), and mixed (a combination of adhesive and cohesive failures). Figure 1 shows the schematic representation of specimen preparation.

## 3. Results

As a result of the 2-way ANOVA test, when the dentin bonding values of two different dentin surfaces and 4 different adhesive systems were compared, a statistically significant difference was observed between the dentin surfaces (superficial and deep dentin) and adhesive systems (*p* < 0.05) (Table 2).

In all adhesive systems, the bond strength with the deep dentin surface was found to be higher than that with superficial dentin and the difference in bond strength value was found to be statistically significant (*p* = 0.05).

**Figure 2:**
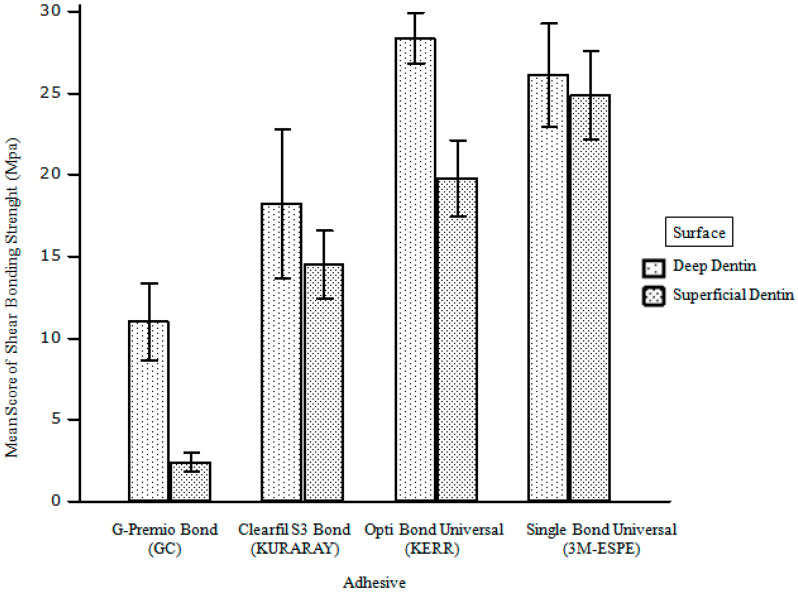
The comparison of the tested groups according to deep and superficial dentin.

When the adhesive systems were evaluated among themselves, the highest bond value to the dentin surface was seen in the Kerr Optibond Universal group (G5) and then in the 3M-ESPE Universal groups (G7, G8) (*p* < 0.05). The lowest bond value to the dentin surface was seen in the GC-Premio group (G2) (*p* < 0.05) (Figure 2) (Table 3).

The difference in bonding value between the Clearfil S3 group and the Optibond Universal group in terms of dentin bond strength with both dentin surfaces was found to be statistically significant (*p* < 0.05). While there was a statistically significant difference in superficial dentin bond strength between the GC-Premio system group and the Clearfil S3 system group (*p* < 0.05), there was no statistically significant difference in deep dentin bond strength (*p* > 0.05) (Table 2).

**Figure 3:**
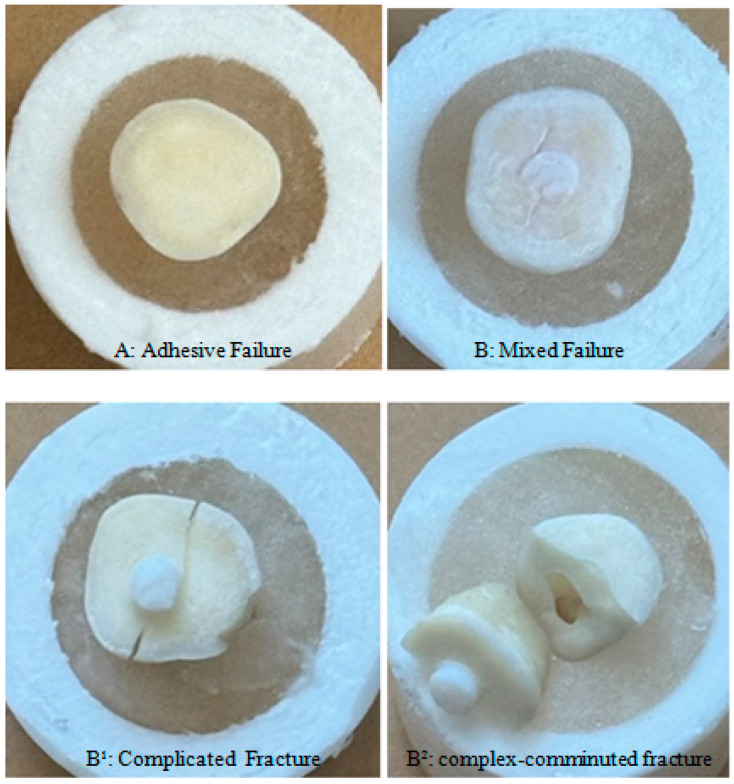
Types of failure. (**A**): adhesive failure; (**B**): mixed failure; (**B^1^**): complicate fracture; (**B^2^**): complex-comminuted fracture. (**B^1^**): complicate fracture; (**B^2^**): complex-comminuted fractures. These were evaluated in the mixed fracture group. Since fractures occurring in teeth are difficult to repair and sometimes teeth can be lost, these results are intended to be shown with the support of figures.

When the bond-break surfaces were examined as a result of the test, 10 (17.9%) adhesive failures and 18 (32.1%) mixed failures were observed in the deep dentin groups (G1, G3, G5, G7). When the superficial dentin groups (G2, G4, G6, G8) were examined, 12 (21.4%) adhesive failures and 16 (28.6%) mixed failures were observed. While adhesive failures were higher in the G-Premio Bond Universal (G1, G2) and Clearfill S3 Bond (G3, G4) groups, mixed failures were higher in the Optibond Kerr (G5, G6) and Single Bond Universal (3M-ESPE) (G7, G8) groups. While no (0) adhesive failures were observed in the deep dentin group (G7) of the Single Bond Universal (3M-ESPE) adhesive, 7 mixed failures were observed. All of these adhesive failures also included tooth fractures. While 1 adhesive failure was observed in the deep and superficial dentin (G5, G6) groups of the Optibond Universal (Kerr) adhesive, 6 mixed failures were observed. Tooth fracture was observed in 5 of the 6 mixed failures in the G6 group (Table 4) (Figure 3).

## 4. Discussion

The adhesive strength of bonding agents to dentin at any depth is theoretically dependent on the area occupied by resin tags, the intertubular dentin area infiltrated by the adhesive resin, and the surface adhesion area [20]. The findings of this study indicated that the shear bond strength values of deep dentin exceeded those of superficial dentin, and this disparity was statistically significant. Consequently, our preliminary concept was rejected.

Burrow et al. indicated in their research that no correlation exists between bond strength and dentin depth [21]. However, certain studies suggested that hydrophilic adhesive systems display reduced attachment strength to deep dentin [22,23]. Our analysis shows that dentin depth substantially affects the bond strength of the completely etch adhesive system to dentin.

This outcome is believed to stem from the utilization of devital teeth and the lack of pulpal pressure under vital circumstances. It is believed that, even without pulpal pressure, deep dentin exhibits significant permeability, and the residual water in the exposed dentin tubules may influence the lateral adhesion of resin extensions [24]. Pashley et al. asserted that when resin extensions are firmly adhered to the lateral walls of demineralized dentin tubules, the bond strength in deep dentin will be equivalent to that in superficial dentin [25].

This study demonstrates that variations in bond strength exist across the evaluated adhesive systems, influenced by the kind of solvent and the pH of the adhesive, which are critical for attaining optimal adhesion to dentin [4].

The solvent type (water, acetone, alcohol, or a combination thereof) differs among adhesive systems. The water content of dentin, or dentin wetness, is a crucial element in attaining good bonding. Gianinni et al. demonstrated that dentin depth influences bond strength values, depending on the bonding region and the adhesive technique employed. The authors indicated that bond strength to medium and deep dentin was inferior to that of superficial dentin. The investigation revealed that the effectiveness of the water-based adhesive method may have been undermined by the elevated water content in deep dentin [26]. Toledano et al. examined the adhesion strength of several bonding techniques to superficial and deep dentin [20]. The highest binding strength values to deep dentin were achieved with a water-based self-etch adhesive containing the functional monomer 10-MDP, alongside acetone-based E and R adhesives. The lowest bond strength values were observed in ethanol-based adhesives [20]. This condition indicates that, among other factors, the kind of solvent is essential for adhesion in various regions of the tooth. Acetone solvent may beneficially affect deep dentin, characterized by increased water content, owing to its designation as the most hydrophilic solvent and its efficacy as a “water chaser” [4]. 

In our study, all adhesives were employed using the total etch technique. The Optibond Universal adhesive exhibited statistically greater bond strength compared to the other adhesive groups. The bond values of the Optibond Universal adhesive group were surpassed by the Single Bond Universal-3M-ESPE and Clearfill S3 Bond Universal groups, respectively. The minimal bond strength value was established in the group employing the G-Premio Universal Bond (GC) adhesive. As a result, our second hypothesis, ‘there is no difference in shear bond strengths of various universal adhesives applied using the etch and rinse (E and R) method to superficial and deep dentin’, was refuted.

Primers or adhesives used on acid-etched dentin include very hydrophilic and diminutive monomers, such as HEMA, which are essential for the hybridization process, effectively wetting and infiltrating the surface [11,27]. The hydrophilic monomer GPDM may operate analogously to HEMA, as it is anticipated to infiltrate the demineralized dentin surface [8]. The effective infiltration of GPDM into extensively exposed collagen accounts for the favorable laboratory and clinical results reported for the 3-step E and R adhesive (OptiBond FL (Kerr)) that incorporates GPDM. GPDM can create a more robust polymer network than HEMA (and MDP) owing to its two methacrylate groups, which facilitate more efficient polymerization with other co-monomers [28]. 

Optibond Universal, in contrast to other universal adhesives, incorporates GPDM as an acidic monomer (10-MDP). In our investigation, as all adhesives were utilized with a comprehensive etch system, meaning the dentin tissue was acidified, GPDM may have diffused more effectively into the collagen fibers exposed in deep dentin. Furthermore, the presence of the ‘acetone’ solvent may exert a beneficial influence on deep dentin, characterized by elevated water content, due to acetone’s status as the most hydrophilic solvent. The aforementioned explanations elucidate the superior adhesion efficacy of Opti-bond Universal adhesive in deep dentin relative to alternative adhesive systems (28.37 MPa).

In our study, the Single Bond Universal adhesive (3M-ESPE) exhibited strong bond strength (G7: 26.12 MPa, G8: 24.88 MPa), comparable to that of Optibond Universal (KERR) (G5: 28.37 MPa, G6: 19.75 MPa). While other adhesives besides Optibond Universal contain 10-MDP, the quantity and purity of MDP may vary within an adhesive system. Prior research has demonstrated that variations in purity and quantity influence adhesive performance [29]. Furthermore, it is believed that another factor contributing to the variation in binding strength values seen in these adhesive systems may be the ratio of 2-hydroxyethyl methacrylate (HEMA) monomer present in their composition. This study employed adhesive formulations containing HEMA, a hydrophilic monomer, in different concentrations. HEMA markedly improves resin penetration into demineralized dentin. However, it has been determined that HEMA negatively impacts the mechanical properties, bond strengths, and polymerization of adhesives [30]. Moreover, HEMA, due to its substantial water retention capabilities, facilitates the hydrolytic degradation of the hybrid layer over time [31]. 

In our investigation, G-Premio Universal Bond, which exhibited the lowest bond strength (G1 (10.99 MPa), G2 (2.41 MPa)), was also utilized using a total etch system. The effectiveness of pre-etching dentin with phosphoric acid before the application of self-etch adhesives is debatable, as acid treatment may impede the resin’s proper infiltration into the etched dentin [9]. This study revealed that pre-etching before the application of G-Premio Universal binding diminished the adhesive’s binding strength. Etching removes high mineral content, hence reducing the availability of calcium for MDP monomer activity [32]. Hosseini et al. (2023) found that the shear bond strength (SBS) of G-Premio Universal Bond adhesive was not significantly different between the SE, E, and R modes when applied to superficial dentin at a pH of 1.5 [32]. The penetration of the resin into the tooth substrate is a factor influencing SBS. Our study diverges from the findings of Hosseini et al. in this regard. Nonetheless, it aligns with the findings of Fabiao et al., where G-Premio Bond was compared to two-stage self-etch adhesives and showed worse bond strength compared to dentin [33]. Based on the findings using the G-Premio Bond, it can be inferred that 10-MDP may enhance adhesion in self-etch applications and that the existence of voids influences bond strength outcomes. In our study, the application of acid to the dentin surface (etch and rinse) followed by the application of a low-pH adhesive (high acidity) (pH = 1.5), such as G-Premio Bond, resulted in a reduction in the calcium necessary for the functionality of the MDP monomer (intense demineralization) and contraction of the dentin collagen. This is believed to be the reason. This condition is believed to elucidate the low-SBS values seen in the G1 group (10.9971 MPa) and particularly in the G2 (2.4100 MPa) group. Functional monomers and purity concentrations in adhesives are reported to diminish bond strength outcomes [34,35]. In the complete etch system, the elimination of the liquid supporting the collagen may lead to the contraction of the collagen network [36,37]. The morphological alterations, especially in deep dentin, may affect the penetration of the applied adhesive into the dentin tubules. The reduction in collagen support may weaken the adhesion to dentin [37]. 

Adhesive failure was predominantly observed in the G-Premio Bond Universal (G1, G2) and Clearfil S3 Bond (G3, G4) cohorts in this investigation. Adhesive failure correlates with diminished shear strength values [38]. Mixed failure was predominantly observed in the Optibond Kerr (G5, G6) and Single Bond Universal (G7, G8) cohorts, elucidating the elevated shear strength values. This failure is prevalent with dentin bonding agents, which chemically interact with tooth structure during monomer infiltration to create a hybrid layer [39]. Mixed-type failures indicate that the experimental composite effectively infiltrated the tooth surface with resin glue. The existence of irreparable fractures in certain samples indicates the potential for tooth survival. Additional scientific research is required to mitigate the risk of irreversible fractures.

## 5. Conclusions

This study revealed that the binding strength of the adhesive systems examined was greater in deep dentin compared to superficial dentin. Optibond Universal and Single Bond exhibited superior bond strength relative to Clearfil S3 Bond and G-Premio Universal Bond in both deep and superficial dentin.

Fewer adhesive failures were noted when the experimental composite utilized in the study (Bioİnfinity Sirius Universal Dental Composite, Avrupa İmplant (Umg Uysal), İstanbul, Turkey) was assessed for detachment from the dentin surface; therefore, Single Bond Universal is recommended for deep dentin, while Optibond Kerr Universal adhesive is advised for superficial dentin.

Additional research is required to optimize the application of the experimental composite with resin adhesive and to minimize tooth fractures.

## Figures and Tables

**Table 1:** Brand names/manufacturers, chemical compositions and pHs of restorative materials used in the study (10-MDP, 10-metakriloyloksidesil dihidrojenfosfat; BIS-GMA, bifenol A glisidilmetakrilat; HEMA, 2-hidroksietil metakrilat; GPDM, glisero-fosfatdimetakrilat; UDMA, üretandimetakrilat; META, metakriloksietiltrimellitatanhidrid; CQ, Kamforokinon; TPO,2,4,6- Trimethyl benzoyl diphenyl phosphine oxide; EDMAB, Etil-4-dimetilaminobenzoat; BHT, bütilhidroksitoluen).

Adhesive System	Manufacturer	Contents	pH
GPremio Bond Universal	Gc Corporatio, Tokyo, Japan	10-MDP, 4-META, 10-MethacryoyloxydecYl Dihydrogen Thiophosphate, Methacrylate Acid Ester, distilled water, acetone, Photo-İnitiators, Silica Fine Powder	1.5
Clearfil S3 Bond Universal	Kuraray, Okayama, Japan	10-MDP, BİS-GMA, HEMA, Hydrophobic Dimethacrylate, Camphorquinone, ethanol, water, Silanated Colloidal Silica	2
Opti Bond Universal	Kerr, Orange, CA, USA	acetone, ethanol, HEMA, Glycerol Phosphate Dimethacrylate (GPDM), Glycerol Dimethacrylate, water	1.9
Single Bond Universal	3m Espe St. Paul, MN, USA	10-MDP, Phosphoric Acid Ester Monomer, Hema, Silane, Dimethacrylate, Vitrebond Copolymer, Filler, ethanol, water, İnitiators, Silane	2.7
**Experimental Composite**			
Bioİnfinity Sirius Universal Dental Composite	Avrupa İmplant (Umg Uysal), İstanbul, Türkiye	BisGMA [%5–10], UDMA [%5–10], BisEMA [%10–15], Filler [%70], Photoinitiator and Stabilizer [CQ [%0.1–0.5], TPO [%0.1–0.5], 4-EDMAB [%0.1–1], BHT [%0.1–0.5] ]	

**Table 2:** Statistical significance between the adhesive system and the dentin surface of the tested groups. DF: degrees of freedom in the source. * comparison only relative to each other.

	Sum of Squares	DF	Mean Square	F-Statistic	*p*-Value
Adhesive system	3126.15	3	1042.05	21.207	<0.001
Dentin surface	430.90	1	430.90	8.769	0.005
Adhesive system * dentin surface	141.79	3	47.26	0.962	0.418

**Table 3:** Mean values of test groups according to deep and superficial dentin groups (MPa).

Adhesive	Surface	n	Mean	Std. Error
GC bonding agent	G1	7	10.9971	2.6494
	G2	7	2.4100	2.6494
Clearfil S3 bonding agent	G3	7	18.2286	2.6494
	G4	7	14.4871	2.6494
Kerr Optibond agent	G5	7	28.3757	2.6494
	G6	7	19.7529	2.6494
3M-ESPE (Single Bond Universal) bonding agent	G7	7	26.1243	2.6494
	G8	7	24.8843	2.6494

**Table 4:** The comparison of the number and percentage of samples of post-fracture failure types of all tested groups (n = 7).

			Mode of Failure (%)			
Adhesive System Name	Type of Dentin Surface	Group Name	Adhesive	Cohesive	Mixed	Total (%)
G-Premio Bond Universal	Deep	G1	5 (71.4%)	-	2 (28.6%)	100%
	Superficial	G2	4 (57.1%)	-	3 (42.9%)	100%
Clearfil S3 Bond Universal	Deep	G3	4 (57.1%)	-	3 (42.9%)	100%
	Superficial	G4	5 (71.4%)	-	2 (28.6%)	100%
Opti Bond Universal	Deep	G5	1 (14.3%)	-	6 (85.7%)	100%
	Superficial	G6	1 (14.3%)	-	6 (85.7%)	100%
Single Bond Universal (3M-ESPE)	Deep	G7	0 (0%)	-	7 (100%)	100%
	Superficial	G8	2 (28.6%)	-	5 (71.4%)	100%
Total			22	0	34	56

## Data Availability

The data presented in this study are openly available in [FaceBase] [https://www.facebase.org] [RRID:SCR_005998].
